# Challenges to the delivery of clinical diabetes services in Ghana created by the COVID-19 pandemic

**DOI:** 10.1177/13558196221111708

**Published:** 2022-07-04

**Authors:** Eunice Twumwaa Tagoe, Justice Nonvignon, Robert van Der Meer, Itamar Megiddo, Brian Godman

**Affiliations:** Management Science, 3527 University of Strathclyde, Glasgow, SL, UK

**Keywords:** diabetes service delivery, COVID-19, Ghana

## Abstract

**Objective:**

The barriers to delivering clinical non-communicable disease services in low- and middle-income countries have risen with the onset of COVID-19. Using Ghana as a case study, this article examines the changes COVID-19 has brought to diabetes service delivery and considers policy responses to deal with future such outbreaks.

**Methods:**

We conducted 18 interviews between November 2020 and February 2021 with health professionals and administrators from primary, secondary and tertiary facilities within the Ghana Health Service. The analysis was performed using deductive and inductive methods.

**Results:**

There were six general themes in interviewees’ responses: (1) COVID-19 had exacerbated the problems of high medicine and service costs and medicine shortages, (2) the pandemic had exacerbated problems of poor patient record keeping, (3) COVID-19 had reduced the availability of suitably trained health providers, (4) staff had become demoralized by management’s unwillingness to make innovative changes to cope with the pandemic, (5) COVID-19 led to a reorganization of diabetes services, and (6) the country’s national health insurance scheme lacked flexibility in dealing with the pandemic.

**Conclusions:**

Access to resources is limited in LMICs. However, our study highlights practical policy responses that can improve health providers’ response to COVID-19 and future pandemics.

## Introduction

The prevalence of adults living with diabetes is increasing globally, especially in low- and middle-income countries (LMICs). There are more than 400 million people with diabetes (PWD) and this is expected to rise to 700 million people in 2045 unless addressed, with middle-income countries contributing approximately 551 million cases.^1^ Middle-income countries have four times more cases than do high-income countries.^1^

Almost 90% of the world’s diabetes-related mortalities occur in LMICs.^1^ Overall, approximately three in every four PWD live in LMICs. Most are unaware of their diabetes status, which means they are never diagnosed or are only diagnosed when complications occur.^1^ Consequently, diabetes prevalence projections may be underestimated. In addition, the calculations do not account for the effect of multiple waves of COVID-19 on diabetes risk distribution and non-communicable disease (NCD) service delivery. Consequently, LMICs will likely face a much greater diabetes burden than previously anticipated, which urgently needs to be addressed to reduce future morbidity and mortality.^1^

Before the COVID-19 epidemic, researchers had identified a number of diabetes service delivery challenges in LMICs. These included high treatment costs, frequent medicine shortages and few trained service providers.^2^ Hospitalizations and medicines are major contributors to diabetes treatment costs in LMICs, and individuals are likely to bear a substantial portion of these costs. This is seen in Nigeria, where studies have shown that the cost of medicines to treat PWD can range from 72 to 90% of total costs, much of which will be out-of-pocket.^2^ For countries that import diabetes medicines—such as Ghana—tariffs and import taxes raise the price of treatment, along with cumulative markups, further reducing their affordability.^3^ These issues are reflected in a wide variation in insulin prices among LMICs, and markups on insulin prices could exceed 500%.^3^ Additionally, access to insulin and diabetes-trained health providers can be limited in LMICs, especially in rural communities.^3^ Strategies have gained policy attention in some LMICs to address the high prices and unavailability of diabetes medicines. These include improving drug-supply chains, prioritizing cheaper and efficacious biosimilars, increasing competition in the pharmaceutical industry and continuous training.^4^

Studies have examined the association between COVID-19 and diabetes. One study found the manifestation of diabetes and its complications (e.g. diabetic ketoacidosis and hyperosmolarity) in previously non-diabetic COVID-19 patients.^5^ Other researchers have investigated the effect of COVID-19 control measures on diabetes treatment. One reported that disruptions from the epidemic – for example, health service disruptions and lockdown - contributed to worse diabetes outcomes.^6^ These build on the concerns of the WHO and others that during the pandemic the health outcomes of PWD could worsen.^6^ Solutions suggested here included teleconsultations with occasional home visits from community health workers, home-delivered medicines and food parcels.^6^

But these studies typically do not examine service providers’ perspectives, nor do they focus exclusively on LMICs – many of which lack the resources available to work around lockdown restrictions when delivering diabetes care. Our study builds on the earlier research by directly interviewing service providers in Ghana to gather evidence on the impact of COVID-19. Our findings will be the first step for clinicians and policymakers in LMICs to design interventions to improve NCD service delivery pathways, as health systems rebuild after COVID-19.

## Methods

### Study design and setting

Ghana first recorded cases of patients with COVID-19 in March 2020. From April, the government implemented a 3-week lockdown in Accra (the country’s capital and largest city) and Kumasi (another large city) and its environs. Hospital outpatient services, including diabetes clinics and other NCD services, were closed during the lockdown.^7^

One of the authors (ETT) interviewed healthcare professionals and administrators in all three levels of care (i.e. primary, secondary and tertiary). The interviews were conducted between November 2020 and February 2021, when many outpatient services, including diabetes clinics, had resumed operations. Interviews were semi-structured, using a topic guide that addressed issues such as service organization, staffing, referrals, patient concerns, data management and health insurance. The topic guide was developed from a literature review conducted for this study and piloted with interviewees in private health facilities in Ghana. The topic guide is available in the online supplement.

### Health facilities and interviewees

Introductory letters were sent to a range of public facilities, which were purposively sampled.^8^ These facilities were located in the Greater Accra region, the only area accessible to the interviewer due to COVID-19–related travel restrictions. Two primary and one secondary facility granted permission to interview clinicians. Through a snowballing technique,^8^ interviews in a tertiary facility were also conducted.

The two primary facilities are in different municipal districts, providing outpatient diabetes services twice weekly to a mix of rural and urban communities. The secondary facility provides secondary to tertiary level care to a large population, serving as a referral point for facilities in the region. Diabetes clinics for outpatients are organized twice weekly. The tertiary facility is a referral facility and provides comprehensive diabetes care, with outpatient diabetes clinics running every weekday.

Ghana has a government-introduced national health insurance scheme, the NHIS. Funded by various levies and other income sources, it ensures Ghanaian residents have access to affordable health care. Patients who receive health care from NHIS-accredited providers can often have their care at least partially paid for by the insurer.^9^ All of the facilities in our study, barring one of the primary facilities, were NHIS-accredited.

In total, 18 interviews were conducted – eight at primary facilities, four at secondary and six at tertiary. After this, no further interviews were conducted due to data saturation (i.e. we received similar responses for the same questions). The interviewees had various administrative and clinical roles. These included physicians, nurses, a physician assistant, a laboratory technician and records officers from the Ghana Health Service. The rationale for including staff from these various roles was to represent the perspectives of clinicians and others providing different diabetes services, in order to paint a comprehensive picture of activities that ensued along the service delivery pathway during the pandemic. Table 1 shows the characteristics of the study participants.

**Table 1.** Characteristics of study participants.

Participant number	Role	Facility type
P1	Chief nursing officer	Secondary
P2	Pharmacist	Tertiary
P3	Physician	Tertiary
P4	Nurse	Tertiary
P5	Chief nursing officer	Tertiary
P6	Nurse	Secondary
P7	Laboratory technician	Tertiary
P8	Physician	Secondary
P9	Records officer	Primary
P10	Pharmacist	Tertiary
P11	Records officer	Primary
P12	Physician assistant	Primary
P13	Nurse	Primary
P14	Dietician	Secondary
P15	Records officer	Primary
P16	Hospital administrator	Primary
P17	Nurse	Primary
P18	Nurse	Primary

All interviews were conducted face-to-face in health facilities with strict adherence to social distancing and other COVID-19 protocols. Open-ended questions were asked, followed by probes for clarification when required.

Interviews lasted 30–40 mins on average and were audio-recorded using a smartphone. Interviews were conducted in English, transcribed, and sent to interviewees to ensure their perspectives were accurately represented. All transcribed interviews were securely stored and recordings deleted.

Ethics approval for the study was granted by the ethics committee of the University of Strathclyde and the Ghana Health Service Ethics Review Committee.

### Data analysis

The study adopted a positivist approach to data analysis. Data were analysed using a hybrid approach of deductive and inductive coding. The researchers applied an a priori template of codes to the data, identified through a literature review conducted for this study. Four broad deductive codes – few diabetes-trained providers, high treatment costs, medicine shortages and low provider motivation – were developed. These were applied to all transcripts, while maintaining an open mind to identify different codes. Four additional inductive codes were identified from the transcripts – high cost of laboratory/service, suboptimal patient information management systems, service organization challenges and health policy–related challenges.

## Results

This section presents the study’s findings under six themes: high medicine and service costs and medicine shortages, poor patient information management, few trained providers, low provider motivation, service organization challenges and national health policy–related concerns.

### High medicine and service costs and medicine shortages

Respondents at all levels of care said the challenge of limited access to medicine and laboratory services in their work had worsened because of COVID-19. They explained that the pandemic has disrupted the supply chain, causing rationing of medicines and reagents in the manufacturing countries and frequent stockout in their facilities. The NHIS continued to pay for insulin, metformin and glibenclamide, but other medicines, such as those for treating diabetic retinopathy, were not covered and consequently cost the patient comparatively more, causing concern. This is because many of the patients are self-employed traders or dependents of traders and they could not engage in trading due to lockdown. Consequently, these medicines have to be paid for out of dwindling savings or just not purchased. One nurse explained:

All our eye drugs, NHIS do not provide for clients, so patients pay for all our eye care services except the consultation fee, which NHIS pays. For the drugs, because most of them are imported, they are expensive…about 65% of clients are not able to afford drugs. (P4).

### A physician said:

Sometimes, you see a patient and he would benefit from one or two things…but because of the cost, you have to give them the cheaper option, which may not be the ideal thing that will help. (P3).

As part of its efforts to control the spread of COVID-19, the Ghanaian government closed its national borders. As most reagents used in laboratory testing are imported, interviewees at secondary and tertiary facilities said the border closure limited access to most of the reagents used in laboratory tests for PWD. When there were no reagents, providers referred patients to private laboratories, most of which were not NHIS-accredited, meaning patients had to pay out-of-pocket, increasing the financial burden on patients.

Interviewees said the pressure on health facilities due to the surge in COVID-19 patients had led them to ration care to existing patients, such as those with diabetes. This contributed to review schedules for prescription drugs lengthening from 1 month to about 3 months. This increased the cost to patients because the NHIS only covered about a month’s prescription. Patients had either to pay out of their own pockets for the remaining medicines or return to health facilities for a dated and signed copy of the same/initial prescription to obtain medicines under NHIS.

Some participants said that, to help patients cope with such increased costs, their health facility offered its own social welfare system. The hospitals’ social welfare systems are usually funded by local and international donors. One pharmacist explained:

‘The attending physician has to declare the patient unable to pay and refer him/her to social welfare.’ (P2).

### Poor patient information management

Interviewees reported that, to avoid close contact with patients who could be infected with COVID-19, providers temporarily stopped measuring patients' weight and blood glucose. In other cases, patients were made to stand on weight scales while wearing footwear to prevent direct bodily contact with measuring scales. Interviewees acknowledged that this could lead to inaccurate weight measurements but said the situation was necessary.

Participants said issues of missing paper folders and identification cards, incomplete entries and illegible writing in records had worsened since outpatient services had resumed. This was as a result of the haste in which health workers had to create free space to serve the increasing number of cases attending after lockdown. Patients, on the other hand, asked known contacts working in hospitals to keep their medical folders in their personal lockers so that they can quickly access them and be seen earlier on their next visit. However, these folders are easily misplaced. According to interviewees, providers could forget critical medical information about patients' conditions because the information had been written in misplaced folders. Where patients’ folders were not available, physicians had to rely on prescription records or patient’s descriptions of their own medicines, which is not ideal. A physician assistant said:

[Patients] mostly come with the medications they take - the boxes - so you are able to at least get the patient and the past medical history and the drug history. (P12).

### Few trained service providers

During the lockdown diabetes clinics and outpatient department services were temporarily halted to allow diabetes-trained doctors and nurses to attend to the high numbers of COVID-19–infected patients. Respondents said they found the situation frustrating because they could not properly manage and treat their diabetes patients during lockdown. One physician complained:

We have to start all over again with the patients because most of them return after the lockdown with uncontrolled sugars…probably because they were eating a poor diet, or they were stressed, and not exercising. (P8).

Interviewees said that, as there were few trained diabetes service providers and resources in primary health facilities, they frequently referred patients to secondary and tertiary facilities. However, patients hesitated to go to higher facilities for fear of contracting COVID-19, and so either received no treatment or sought treatment elsewhere. A physician shared their experience of a 16-year-old patient with high blood sugar:

She had been to a private clinic…they gave her metformin. Maybe the person [at the private clinic] does not know that high sugar in the child is not type 2 diabetes, it is likely type 1, and that person will need insulin. So, the patient's time and money were wasted on metformin. The day she came here she was in DKA [diabetic ketoacidosis], so she had to be admitted (P3).

### Low provider motivation

Interviewees in secondary and tertiary facilities said other providers and hospital management had not been receptive to new ideas to improve service delivery during the COVID-19 pandemic. Interviewees said strategies had been devised to reschedule appointments to prevent facilities becoming overcrowded with COVID-19 patients and thus allow clinicians to treat diabetes patients and others. But management responded with indifference. Participants found this demoralizing, as a nurse explained:

[They] simply do not want to change, because they have been here for a long time and that is exactly what they have been doing all the time. (P6).

### Service organization challenges

Participants said that COVID-19 had led to a reorganization of diabetes services. The usual monthly reviews of patients had been extended to 3 months to limit patients’ risk of contracting COVID-19 due to frequent hospital visits. But this could have adverse effects. A prolonged time between reviews limited opportunities for providers to reemphasize healthy behaviours and intervene early in patients’ conditions to prevent diabetes complications. As one physician said:

If I see a patient whose sugars are not controlled, ideally, the next review should be closer. But now this is very difficult. The COVID allows us to see a certain number of patients a day, so we cannot see so many patients. So, you realize that the clinic’s dates are longer intervals than usual. So, control is very difficult. (P8).

Interviewees said that between appointments they had no way of checking up on patients. During those intervening periods some patients listened to ill-informed advice on managing their high blood sugars, engaged in unhealthy behaviours, and ended up in hospital with severe illness. A physician said:

I wish we had a telephone service, where, if a patient is at home and review is in the next three months…and if he has a challenge, he can be able to call for guidance before review time is due. It is something that, if we had, it would help because, for all you know, they may be at home receiving misguidance from friends. Sometimes if the patient is in contact with the health care worker who can give the correct advice, it will help our management. (P3).

Another problem was overcrowding and long waits at clinics. According to interviewees, patients crowd outpatient departments from early morning, hoping to be seen early so that they can then go to their workplaces. However, this is not always possible because doctors attend to inpatients before seeing outpatients. Respondents raised concerns about COVID-19 spreading during diabetes clinics due to such overcrowding, as well as some patients’ disregarding safety protocols (e.g. wearing a face mask). To help address this issue providers reduced the number of patients scheduled per clinic day. A records officer said:

We need to expand in terms of physical space. Our consulting rooms are not many and the wards are getting full. We are trying to obtain funds to erect more buildings but that has not been easy. The diabetes clinic has only two consulting rooms and since we resumed from the lockdown, on Tuesdays and Fridays the place is always full, with long queues and some patients don’t even wear nose masks. (P15).

### National health policy–related concerns

Participants said that to standardize medicine prices, the NHIS is against facilities selling covered medicines to insured patients. Even when medicines are stocked out in the central medicine store, health facilities cannot buy medicines on the open market without the central medical stores’ permission. Participants explained that after purchasing from the open market, hospitals should not sell medicines to patients at 15–20% more than the NHIS prices. Participants said these restrictions contributed to a shortage of diabetes medicines during the COVID-19 pandemic because they could not readily buy limited available medicines on the market without the central store’s permission. According to interviewees, obtaining permission can be a slow and complex process. During the wait, treatment is delayed, and patients could suffer complications or death due to unavailable medicines, especially with insulin shortages. As one nurse said:

If there is any delay in procurement from the central point, you do not have the liberty to go to the market to procure [medicine]. So then we run out of a lot of vital or essentials medicines, like paracetamol, metformin, glibenclamide. (P4).

Additionally, the NHIS does not allow for the sharing of medicine costs with insured patients and doing so can attract disciplinary actions from the National Insurance Authority. An administrator explained how concerns about patient cost-sharing led to his facility being declined NHIS accreditation:

NHIA had to pay us more, but they were not willing to, and they did not want us to charge patients for the difference in payment. So, we quitted. (P15).

Respondents explained that if patients are made to pay the price difference after deducting the NHIS reimbursement price, there would be less incidence of treatment delays due to unavailable medicines as facilities will source from the open market with fewer price restrictions.

## Discussion

This study described service providers’ perspectives on the changes COVID-19 brought to diabetes service delivery in public health facilities in LMICs, using Ghana as a case study. We highlighted numerous adverse effects, but there are a number of policy strategies that could be used to help mitigate these.

Respondents stated that the high cost of non–NHIS-funded medicines impeded effective diabetes treatment because patients could not afford these medicines. Ghana has identified the high cost of medicines as a general problem and has instituted the National Medicine Policy to enhance the operations of the pharmaceutical sector (drug manufacture, procurement and pricing).^10^ However, the challenge with unstandardized medicine prices remains in Ghana and other LMICs, with the cost of diabetes medicines ranging from USD15 to over USD500 per year.^3,10^ The price of diabetes medicines has also increased in high-income countries. For instance, in England there has been a 17% increase in the total cost of diabetes medicines over the past 5 years.^11^ Steps to increase standardized medicine prices should include negotiations between key stakeholders (e.g. government, pharmaceutical companies and consumers) to determine price floors/ceilings that will ensure fair prices for both manufacturers and patients.^12^

Respondents noted that the lockdown and the closure of diabetes clinics during the pandemic contributed to greatly reduced availability of diabetes services. While similar findings are reported in many countries,^13^ some nations developed innovative responses to the problem. The United Kingdom, for instance, developed strategies to deliver diabetes care during the pandemic, including posting urinalysis dipsticks to type 1 diabetes patients, who self-tested and then uploaded the results to a mobile phone application, which transferred the information to healthcare providers.^14^ Further, in Scotland, online diabetes support groups for PWD were formed to support health promotion and education delivered remotely.^14^ In Italy, health providers use Facebook, video teleconsultation and websites to deliver diabetes services.^13^ In India, teleconsultations using trained pharmacologists has proven to be an effective to deliver diabetes care.^15^ Drones were even used in some parts of Africa to deliver medicines during the pandemic.^14^ While we are aware that resource constraints may limit the implementation of similar strategies in LMICs, at least some of these technologies could be used to deliver health services to populations during restrictions such as those imposed during the COVID-19 pandemic.

Participants said that both service providers’ haste to attend to queued patients and patients’ attempts to quickly get their folders to see a doctor contributed to missing records and incomplete and inaccurate patient information. Studies have reported growing concerns about the quality of routine health information systems data in LIMCs, necessitating the intermittent use of cross-sectional national surveys to collect data.^16^ However, reforms are occurring. The District Health Information Management Systems, platforms for organizing population-wide health data, have helped improve health information management in LMICs.^17^ Ghana’s web-based version collects data on services delivery, facility resources and public health activities, and the data is accessible for research and practice.^17^

Respondents emphasized that, after the lockdown, diabetes clinics were overcrowded and patient waiting times lengthened. This increased the likelihood of patients and providers becoming infected with COVID-19 in overcrowded diabetes clinics. Addressing these issues requires a redesign of appointment scheduling and record keeping.^18^ Changes in hospital equipment can also help. For instance, the use of fans instead of air conditioners to improve air circulation and ventilation helps reduce the risk of infection in hospitals and other enclosed public places.^19^

COVID-19 has reaffirmed the need for more trained health professionals throughout the world, including in LMICs, to avoid the anticipated shortage of 15 million people in the global health labour market by 2030.^20^ Interviewees described how the surge in COVID-19 patients saw the already few trained diabetes service providers having to switch to treating COVID-19 patients. As well as training more health professionals, existing staff can be redeployed. In India, for instance, the use of trained pharmacists to replace doctors in providing diabetes follow-up consultations during the COVID-19 epidemic proved viable and effective.^15^ Likewise, other non-physician healthcare providers such as physician assistants and nurses can pick up some roles of doctors in the delivery of NCD services, if they are provided with adequate training and continuous learning. For example, physician assistants and nurses have been reported to substitute or supplement physicians’ roles in delivering diabetes care to adult in the US.^21^

Alongside this, participants mentioned that inadequate material and human resources in primary care, coupled with the lack of recognition and appreciation of providers in secondary and tertiary facilities, reduced their motivation to work. Working conditions, financial and social incentives, and career development are commonly reported to influence health providers’ motivation.^22^ Interventions focused on continuous education and mentorship can improve provider knowledge and skills in the short term. However, the level of performance improvement differs depending on the nature of the task and the cadre of health worker.^23^ Policymakers should investigate what motivates health workers at different levels of care to inform the design of tailored interventions.

To reduce overcrowding in clinics and the potential for increased COVID-19 infection rates, providers have reduced the number of patients scheduled per clinic day and increased the time between prescription review appointments from one month to about three. This strategy has possible negative implications for diabetes outcomes, including missed opportunities to reiterate healthy behaviours and to instruct patients on how to take medicines appropriately. Consequently, PWD were likely to report poorly controlled blood glucose and severe illness as a result of the pandemic.^24^ One could counter that a study conducted in Turkey did not find a significant difference between average blood glucose measurements in people with type 2 diabetes after the COVID-19 lockdown period.^25^ However, this could have been because patients had less stress in their working lives due to working at home, had more time to concentrate on lifestyle needs, had access to digital technologies to help monitor insulin levels, and had regular virtual consultations.^24^ This is quite a different set of circumstances to PWD in LMICs, who are struggling with a lack of available resources and high co-payments.^22^ To make matters worse, many patients could not engage in economic activity during the lockdown, which significantly increased household poverty and lowered peoples’ living standards.^26^

Interviewees said that the cost-sharing prohibition under the NHIS contributed to medicine shortages and treatment delays for PWD. Cost-sharing saves individuals from catastrophic health expenditures and can raise funds for the NHIS.^27^ If the NHIS had introduced a cost-sharing scheme where patients pay 5% of their treatment cost before the pandemic hit (in the period 2007–2015), it would not be in any difficulty funding its operations now since the scheme would have had excess operational funds; however, the health service utilization rate under the NHIS would have reduced by 15%.^27^ Policymakers should consider redesigning diabetes service coverage in the NHIS.

Finally, participants noted that the persistent medicine shortage and limitations on health facilities to procure and price medicines delayed patients’ access to diabetes medicines. In Ghana, only a few health facilities (mostly those in urban areas) have a stock of essential medicines for PWD.^3,4^ Globally, three multinational companies – Novo Nordisk, Eli Lilly and Sanofi – dominate insulin production. These companies control 96% of the global insulin supply, and the failure of governments to outsource from different manufacturers means medicines get stockout if these companies do not meet demand.^28^ The WHO prequalification programme, whereby the WHO prequalifies the quality of biosimilar insulins,^29^ could help increase access to insulins and other key medicines by encouraging through increasing competition low-priced diabetes medicines and equipment that meet agreed quality, safety and efficacy standards. This is because the WHO initiative programme prequalifies pharmaceuticals and diagnostics that satisfy international standards through multiple assessment methods, discovering and correcting quality concerns and boosting quality assurance. In addition, the programme encourages global competition for high-quality medications and diagnostics. Ghana and other LMICs should also work with drug manufacturers building on interventions such as the Base of the Pyramid initiatives in Nigeria, Tanzania and Kenya^30^ to ensure diabetes medicine is accessible. Ghana’s Ministry of Health should seek to reimburse low-cost biosimilar insulins listed on its essential medicine list (a collection of pharmaceuticals derived from the Ghana Standard Treatment Guidelines to ensure consistency in treatment, procurement and reimbursement),^10^ including those that are increasingly prequalified by the WHO, to ensure best value for money through increasing competition.^4^

### Limitations

The study has three main limitations. First, in response to the pandemic, health facilities continue to change how health services are organized and delivered. As such, the findings of this study may not reflect present circumstances. Second, the interviews were conducted in open workspaces within health facilities. This may have reduced interviewees’ level of candour.

Third, we were not able to interview diabetes service providers outside the Greater Accra region or those in facilities that remained closed to outpatient diabetes care. Furthermore, we did not canvas the views of PWD. As such, this study only gathered a limited number of perspectives.

## Conclusion

The study raises awareness about COVID-19–related challenges in service delivery to PWD in LMICs. While access to resources in such countries is, of course, limited, our study has highlighted practical policy responses that can improve health providers’ responses to COVID-19 and future pandemics.

## Supplemental Material

Supplemental Material – Challenges to the delivery of clinical diabetes services in Ghana created by the COVID-19 pandemic Click here for additional data file. Supplemental Material - Challenges to the delivery of clinical diabetes services in Ghana created by the COVID-19 pandemic for Eunice Twumwaa Tagoe, Justice Nonvignon, Robert van Der Meer, Itamar Megiddo and Brian Godman in Journal of Health Services Research & Policy
