# Supplementary material for: Challenges to the delivery of clinical diabetes services in Ghana created by the COVID-19 pandemic
Source: J Health Serv Res Policy. 2022 Jul 4;28(1):58–65. doi: 10.1177/13558196221111708 (PMC9253720; doi:10.1177/13558196221111708)
Supplement: Supplemental Material – Challenges to the delivery of clinical diabetes services in Ghana created by the COVID-19 pandemic [file sj-pdf-1-hsr-10.1177_13558196221111708.pdf]

Journal of Health Services Research & Policy
Challenges to the delivery of clinical diabetes services in Ghana created by the COVID-19 pandemic
Tagoe ET, Nonvignon J, van Der Meer R, Megiddo I, and Godman B

Supplement 1: Topic guide for interviews – Challenges to the delivery of clinical diabetes services in Ghana created by the COVID-19 pandemic

[Assume participants have read PIS and so have a background to the study, and have completed consent]

Access to diabetes services

Can you tell me about your role and expertise with diabetes service delivery and policy decision-making? Can you tell me about the diabetes services your facility provides?

Can you explain the routes patients could use to seek care in your facility? What are the gatekeeping requirements for accessing diabetes care in your facility? How has this route changed since the COVID-19 epidemic?

Diabetes service organisation

Can you briefly describe the procedures involved in diabetes services delivery in your facility? Can you tell me how these services are organised (e.g., clinic days and scheduling appointments and reminders and patients' data management)? Can you briefly tell me which procedure you are primarily involved with and the challenges you encounter organising and delivering diabetes services? How have these challenges evolved with COVID-19? Can you tell me about any other challenges your colleague providers encounter while providing diabetes care?

Medicines and supplies

Can you tell me about how your facility obtains medical supplies and drugs for diabetes care? Can you briefly describe the challenges your facility encounters with medical supplies and

diabetes medicines? How has the COVID-19 epidemic affected the supply of medicines and logistics you require to deliver diabetes services? How do these challenges affect your work?

Diabetes service referrals

Can you briefly explain when and how your facility refers people with diabetes to other hospitals/ resources? Can you tell me about any referral challenges and how that affects the delivery of diabetes care? Can you tell me how COVID-19 has affected the way your facility refers patients to other health facilities?

Service provider expertise

Can you tell me about the level of staffing for diabetes care in your facility and how the COVID-19 epidemic influences the staff available to provide diabetes services? What or which expertise would be needed for sufficient staffing?

Diabetes service delivery cost

Can you tell me how patients pay for the diabetes services your facility provides? Can you tell me briefly what diabetes services/procedures/medication most patients cannot afford? Can you tell me how your facility manages patients who cannot pay for services? Can you tell me how the COVID-19 epidemic influences patients' ability to afford diabetes services? How does this challenge affect their treatment outcome?

Are there any other challenges we have not discussed that you would like to mention?

What do you recommend for improving diabetes service delivery in Ghana?
